# Implementation and Evaluation of a Web-Based Distribution System For Anesthesia Department Guidelines and Standard Operating Procedures: Qualitative Study and Content Analysis

**DOI:** 10.2196/14482

**Published:** 2019-08-15

**Authors:** Kaspar F Bachmann, Christian Vetter, Lars Wenzel, Christoph Konrad, Andreas P Vogt

**Affiliations:** 1 Department of Anaesthesiology & Pain Medicine Inselspital, Bern University Hospital University of Bern Bern Switzerland; 2 Department of Anaesthesiology & Pain Medicine Kantonsspital Lucerne Lucerne Switzerland

**Keywords:** standards, computer communication networks, anesthesiology, decision making, computer-assisted

## Abstract

**Background:**

Digitization is spreading exponentially in medical care, with improved availability of electronic devices. Guidelines and standard operating procedures (SOPs) form an important part of daily clinical routine, and adherence is associated with improved outcomes.

**Objective:**

This study aimed to evaluate a digital solution for the maintenance and distribution of SOPs and guidelines in 2 different anesthesiology departments in Switzerland.

**Methods:**

A content management system (CMS), WordPress, was set up in 2 tertiary-level hospitals within 1 year: the Department of Anesthesiology and Pain Medicine at the Kantonsspital Lucerne in Lucerne, Switzerland, as an open-access system, followed by a similar system for internal usage in the Department of Anaesthesiology and Pain Medicine of the Inselspital, Bern University Hospital, in Bern, Switzerland. We analyzed the requirements and implementation processes needed to successfully set up these systems, and we evaluated the systems’ impact by analyzing content and usage.

**Results:**

The systems’ generated exportable metadata, such as traffic and content. Analysis of the exported metadata showed that the Lucerne website had 269 pages managed by 44 users, with 88,124 visits per month (worldwide access possible), and the Bern website had 341 pages managed by 35 users, with 1765 visits per month (access only possible from within the institution). Creation of an open-access system resulted in third-party interest in the published guidelines and SOPs. The implementation process can be performed over the course of 1 year and setup and maintenance costs are low.

**Conclusions:**

A CMS, such as WordPress, is a suitable solution for distributing and managing guidelines and SOPs. Content is easily accessible and is accessed frequently. Metadata from the system allow live monitoring of usage and suggest that the system be accepted and appreciated by the users. In the future, Web-based solutions could be an important tool to handle guidelines and SOPs, but further studies are needed to assess the effect of these systems.

## Introduction

### Guidelines and Standard Operating Procedures

Generally, guidelines and standard operating procedures (SOPs) are an integral part of perioperative medicine, and these are particularly an integral part of anesthesiology. They have found their way into daily clinical routine, and they form the basis for patient safety algorithms [1-3]. Adherence to guidelines has been associated with improved outcomes in the fields of anesthesiology and intensive care, and it has an impact on patient safety, employee training, and overall quality [4-7]. Guidelines have also been shown to motivate a team, especially if the employees were involved in creating the content [8]. However, the creation, maintenance, and distribution of these guidelines within an institution can be challenging, and the potential benefits and drawbacks remain unclear [9], especially as scientific data on the benefits of guidelines are scarce. Furthermore, measuring quality and safety in anesthesia remains a challenge [10], especially as there are only a few validated indicators, and evidence of their scientific validity is low [11].

### Digitization in Medical Care

Before the age of computers, many institutions distributed their guidelines and SOPs in paper form. Over time, the increased use and availability of computers has led to the digitization of medical care [12,13]. During this transformation, many printed guidelines were transformed into digital files, often using PDF, and these files were commonly stored on local servers. However, this approach can lead to outdated files, and availability to users (eg, the anesthesia providers) is limited, with no search function or linking of content. Managing and reviewing content is challenging and laborious. In the era of digitization, with increased access to computers in the operating room, a fully computerized approach to this problem seems practical, and improved adherence to guidelines can be expected because of improved availability [14]. Digitization has enabled solutions that provide fast navigation, a broad overview, and new formats for content, such as movies. Digitized learning material and mobile learning, in general, can be effective [15]. With digital solutions, content is easily accessible and can be managed in a centralized database, and updates are easy and time saving. The choice of a content management system (CMS) depends on various factors, such as the publication process, accessibility, open-platform support, the implementation process, costs, and security [16]. Scientific evidence to support the implementation of digital distribution and CMSs remains scarce, and issues, such as insufficient security or unsatisfactory publication processes, have been raised with certain workflows [17].

### Aim

This paper presents a digital solution for the development, distribution, and management of guidelines and SOPs. We have successfully implemented our Web-based solution in 2 different anesthesiology departments in Switzerland. In this paper, we describe the necessary requirements, the implementation process, and the metadata generated by the users (ie, employees of the department). Furthermore, we lay out the process for content management and development.

## Methods

### Content Management Systems

The CMS is a software used to create, update, and organize content produced by a defined group. This is usually done with a Web-based solution. The CMS allows creation and structuring of websites, without advanced knowledge or training in coding. The most common Web-based CMSs are WordPress, Joomla, Drupal, and TYPO3 [18]. Through the front end of the CMS (the interface for the regular user), users can access the content either directly or through a search function and receive information about upcoming updates and news within the system. Content managers can log in to the system and access the back end, through which content is created, updated, and distributed to the users. Administrators are in charge of system updates, as well as troubleshooting. Multiple users can access the CMS simultaneously, and role-based access control maps users to roles and different levels of permission. The goal of the CMS is to make permission management convenient by grouping users into different roles and enabling them to work within the CMS.

### WordPress as a Content Management System

Various CMSs exist, and they can be used to distribute guidelines and SOPs within an institution. We chose WordPress (WordPress Foundation), as it can be implemented independently, is free, can be adapted to individual needs, and is intuitive to use. WordPress is a Web-based app, running on an Apache HTTP Server (Apache Software Foundation) in a Hypertext Preprocessor (PHP) environment (The PHP Group, php.net). It requires database access, such as MySQL (Oracle Corporation) or MariaDB (MariaDB Corporation). In November 2018, WordPress was the most frequently used CMS worldwide (used by 60.7% of all websites whose CMS is public knowledge) [18]. This results in extensive resources and a huge support community.

### Implementation Process

**Figure 1:**
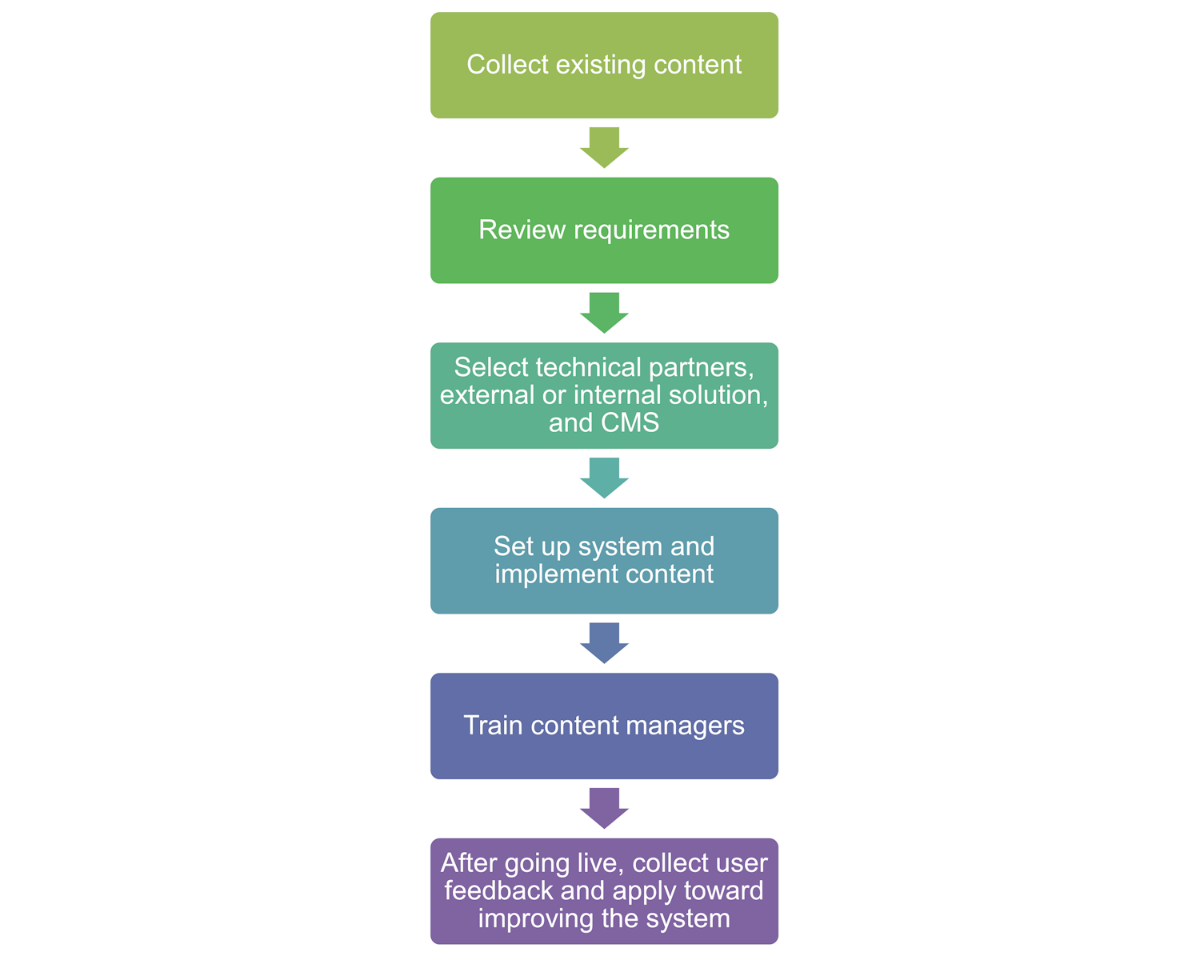
Process of implementing the content management system in an institution. CMS: content management system.

The system’s main purpose is the distribution of general and local SOPs and guidelines. The system will never store any kind of patient-related data. An overview of the implementation process is shown in Figure 1.

Before implementation of the system, the existing guidelines and SOPs were collected to gain an overview of the existing content. After that, we performed a review of requirements (Textbox 1).

Depending on the technical knowledge available within a department, a technical partner for the system implementation may be needed. The system can either be run internally within the institution or externally on a public server that is accessible worldwide. This ultimately depends on the sensitivity of the information published and the technical possibilities within an institution. Both solutions offer advantages and disadvantages (Table 1).

**System requirements.**

Requirements for the back end.

- The system must have a centralized database, which can be accessed through different devices
- The system can be managed and developed simultaneously by different users. Personnel shortages must not impede the performance
- Drafting, commenting, and revising content must be possible before publishing
- All changes and revisions must be trackable
- Data must be backed up on a regular basis
- Updates and maintenance must be performed regularly

Requirements for the front end.

- The user must be able to see which contents were updated and when
- Users must be able to find the desired content using simple navigation or a fast-performing search function
- There should be no limitations as far as devices or operating systems are concerned

**Table 1:** Advantages and disadvantages of external and internal systems

System type	Advantages	Disadvantages
External system	Accessible on all devices at any time Accessible from home and while on the go Publicity for the institution	External costs might apply Data might not be stored internally Security risks might arise, especially if updates are not performed regularly
Internal system	Security and privacy Internal support systems are available	Depending on the technical settings, an internal setup might be complex Content can only be accessed through internal devices. Usually, access from home or on the go is not possible

After we had chosen a hosting platform and decided to develop an internal system (Bern) and an external system (Lucerne), the systems could be set up. Given the open-source character of the software used, documentation was easily available. An operational concept with specified roles and responsibilities was drafted, and this concept was approved by the head of the department. Requirements pertaining to availability and security were defined.

Content managers (ie, users in charge of different sections, such as attending specialists in charge of certain anesthesia divisions) needed training. One of the big advantages of such a system is that content management can be delegated to a number of employees within the department. This precludes a bottleneck that could develop if only a single user or a few users are in charge of content management. Finally, an in-depth analysis of metadata and usage was performed.

### Statistical Analysis and Metadata

Metadata generated by user access were recorded with either WP Statistics (Verona Labs) or Visitor Statistics Pro. Data were imported and analyzed using Microsoft Excel (Version 2016) or Sigmaplot 13.0 (Systat Software). Continuous variables were expressed as means (SD); categorical variables were presented as frequencies and percentages.

## Results

### Implementation and Content

The system was implemented in 2 tertiary-level hospitals in Switzerland: first, it was implemented in the Department of Anesthesiology and Pain Medicine at the Kantonsspital Lucerne in Lucerne, Switzerland, and second, it was implemented in the Department of Anaesthesiology and Pain Medicine of the Inselspital, Bern University Hospital, in Bern, Switzerland. The content was divided into various sections (eg, clinical anesthesia, regional anesthesia, and airway management), and each section was overseen by a senior specialist. This included creating new content, as well as updating existing pages. Content was reviewed at least once a year. Users could report directly to the senior specialist in charge if they noticed a need for changes or for implementation of new content. Content could be navigated using a menu bar or accessed directly through a search function. The most frequently accessed content was guidelines on regional anesthesia, followed by various SOPs used with clinical anesthesia.

### Department of Anesthesiology and Pain Medicine, Kantonsspital Lucerne

**Figure 2:**
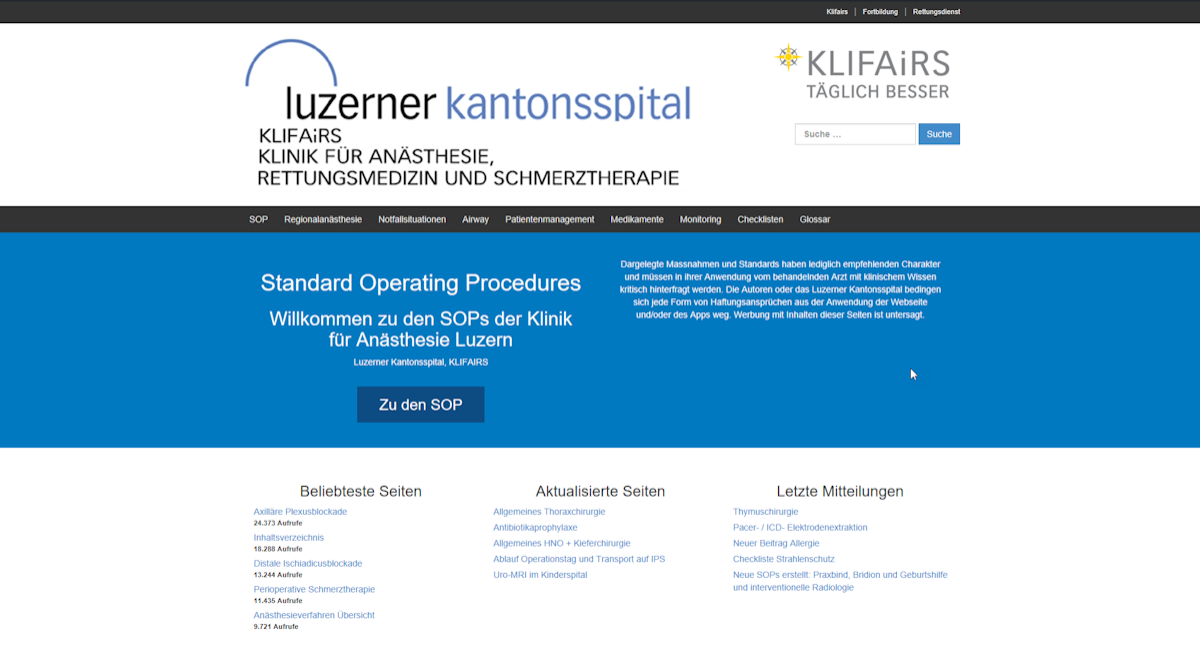
Screenshot of the content management system running in Lucerne.

WordPress was set up on an external server, providing worldwide access to the department’s SOPs and guidelines (Figure 2). The system went live in May 2014. The external server is hosted by a Swiss hosting company, and it provides the necessary infrastructure, such as the latest versions of PHP and MySQL, and it includes a preinstalled version of WordPress. There is no need for manual app setup or updates, apart from WordPress itself. Implementation took place over a period of roughly 1 year. This involved a requirements analysis, the collection of already existing guidelines and SOPs, the setting up of a test website, and the gradual transfer of the content to the CMS. There are 269 pages of content within the CMS. These pages are divided into clinical SOPs (eg, SOPs for neuroanesthesia, cardiac anesthesia; 196 pages), regional anesthesia guidelines (13 pages), emergency guidelines (7 pages), airway guidelines (6 pages), patient management guidelines (eg, patients with diabetes, kidney disease; 18 pages), guidelines on drugs (10 pages), SOPs for monitoring (6 pages), and checklists (13 pages). In the 365 days ending on November 1, 2018, there were 155,379 visitors to the website, corresponding to 1,057,492 website requests. As the content is in German, the website is primarily accessed by people in German-speaking countries (73,136 visitors from Germany, 41,262 visitors from Switzerland, and 9852 visitors from Austria). Access to the website was primarily gained using iPhone (113,692 visitors, 35.80%) or Windows (113,213 visitors, 35.28%; Table 2). There are 44 registered users involved in managing the content. The cost of this setup is minimal. Other than buying a domain name (CHF 70) and paying for a hosting service (CHF 100 per year), there were no financial investments. This did not include the time invested by the department’s employees. Roughly 300 to 400 hours were needed for system setup and 50 hours per year for system maintenance. This resulted in an overall cost of less than CHF 1000 for the entire project. The hosting company updates all server applications on a regular basis. The administrators perform WordPress core updates and updates of all installed plug-ins multiple times per year. This ensures that security flaws are promptly fixed. Furthermore, we ran All In One WP Security (Tips and Trick HQ), which protects the website from unwarranted access, with an additional firewall function. No patient-relevant data were stored on the website. Content was backed up on a weekly basis, and backups were kept for half a year.

**Table 2:** Operating systems most commonly used to access the Lucerne Hospital’s hosting system.

Operating system	Overall use, %
Windows	35.28
iPhone	34.80
Android	7.11
iPad	6.81
Macintosh	11.01
Linux	0.99
Unknown	3.99

### Department of Anaesthesiology and Pain Medicine, Inselspital, Bern University Hospital

**Figure 3:**
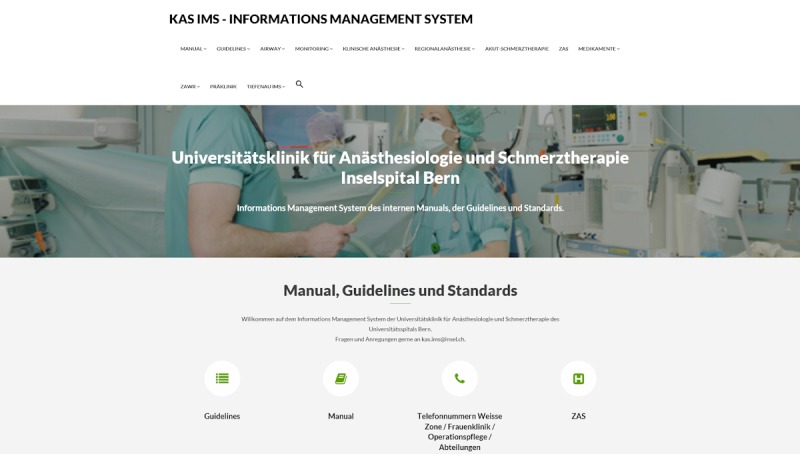
Screenshot of the internal content management system running in Bern.

The system was set up on an internal departmental server and went live in March 2018 (Figure 3). External access is impossible, as the system operates in a separate domain behind the hospital firewall. The infrastructure runs on a Windows Server (Version 2012 R2); Apache HTTP Server, PHP, MariaDB, and WordPress were installed manually. The entire setup process was documented. As installations are maintained manually and the systems need constant development, the setup comprises a test server, as well as a live server. All updates and major changes to the system are first established within the test environment before going live. The project started in March 2017, and realization was possible within 12 months. Content is presented on 341 pages managed by 35 active users. Content is divided into the following sections: in-hospital SOPs (eg, local phone numbers, operating room schedule, and hygiene; 16 pages), clinical anesthesia (236 pages), patient management (18 pages), airway guidelines (6 pages), guidelines on monitoring (3 pages), regional anesthesia (8 pages), SOPs on pain therapy (41 pages), guidelines for drugs (6 pages), and SOPs for postanesthesia care (7 pages).

**Figure 4:**
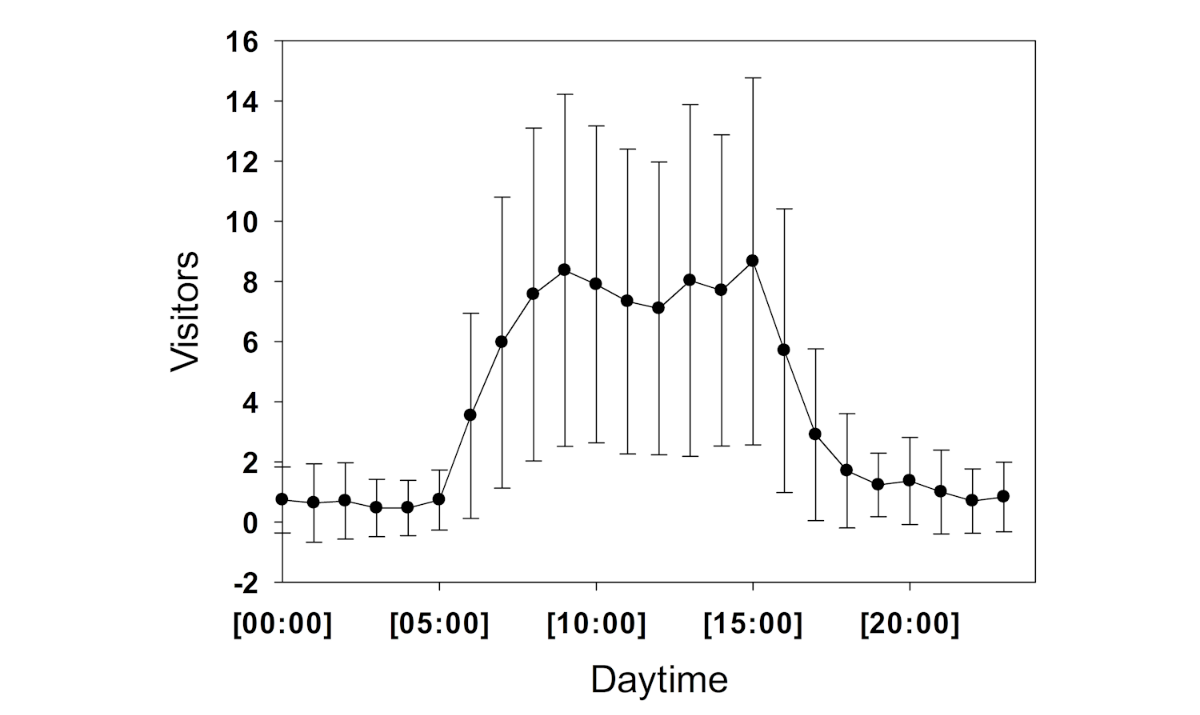
Hourly usage of the internal content management system for October and November 2018 at the Bern University Hospital. Dots represent means and whiskers represent SD.

Since going live, the system has been visited 13,856 times, corresponding to 45,284 page views (3.27 page views per visit). Users access the website predominantly from outside the operating room or patient care, with only 25% of users using computers positioned directly at the anesthesia station and 75 % using computers outside of the operating room. As the system is locked behind the department’s firewall, virtual private network was not provided for mobile phones, and no mobile phone access was possible. Hourly usage peaked twice daily, between 9 am and 10 am, with 8.4 (SD 5.9) visitors, and between 3 pm and 4 pm, with 8.7 (SD 6.1) visitors (Figure 4). As there are no expenses for external servers, the system has not produced any expenses, except for the time which system developers and users dedicated to the platform.

As the system runs on an internal platform with in-hospital access only, digital attacks from the World Wide Web are not possible, and the website is protected by the corporate firewall. However, all apps and plug-ins are updated regularly, which ensures that security holes are closed. The system does not contain any patient-relevant information, and it is backed up daily. System security was discussed with the information technology department.

## Discussion

### Principal Findings

The implementation process described in this paper has been successfully performed twice in large anesthesia departments in Switzerland. Although the process requires some technological knowledge or at least an appropriate technical partner, setup itself is easy, and it can be done within a reasonable period of time. There are various CMS options available, and CMSs have been shown to be an ideal tool for efficient and consistent management of guidelines [16]. Our detailed requirement review allowed us to choose an appropriate CMS and showed that WordPress would be superior to regular wiki-based systems. Metadata generated by the systems show high usage. Use during the day reflects the active hours in our operating rooms. However, as the database is primarily accessed independent of direct patient care, it is safe to assume that its primary use is as a reference book. As a limitation to our study, direct comparison of the systems is not possible, as the Lucerne system is hosted externally, and its metadata are skewed by worldwide access. However, the statistics from Lucerne show that there is international interest in these guidelines and SOPs.

### Content Management Systems in the Health Care Sector and Digital Maturity

WordPress has already been used as an electronic portfolio system [19], as a platform for the dissemination of evidence-based medicine [20], and as a centralized in-hospital database to share and distribute information, with low costs [21]. However, it has never been applied as a distribution system for guidelines and SOPs in anesthesia or emergency care. With WordPress, content can be easily managed by our senior staff, who are able to create and update pages. It is a major advantage that these content managers are anesthesiologists working in clinical practice on a daily basis and are thus in close contact with the users. This ensures a direct feedback loop and prompts implementation of new content or updates to existing content. The possibility of having multiple users manage content simultaneously prevents the system from being dependent on a single person or a small group, as might be the case with an individually coded app (eg, iOS or Android). WordPress is the most commonly used CMS worldwide, appreciated for its flexibility and features. However, this also makes it a target for security breaches and attacks. The best way to protect a system is by always running the latest stable release [22]. To avoid problems, we do not keep any sensitive information, especially patient-related data, in our databases. The modernization of the health care sector in Western Europe was reported to increase spending in technology and informatics from US $13.2 billion in 2013 to US $14.6 billion in 2018 [23]. In an assessment of Swiss hospitals, investment in hardware and software was found to be the most promising way to improve digital maturity of a health care organization [23]. The concept of digital maturity represents the ability to respond to changing needs and challenges in a computerized world. A strong link was found between usage intensity and digital maturity. The implementation of a Web-based CMS might be seen as an investment in software solutions, and therefore, it might be seen as an improvement of the digital maturity of the 2 corresponding health care organizations. It seems reasonable to record usage intensity over time to assess whether these apps might increase digital maturity through wider usage.

### Mobile Technology Enhancing Accessibility and Usability

Our metadata concerning operating systems show that mobile phones are frequently used to access our guidelines and SOPs. Mobile learning has been shown to be as effective as traditional learning [15], suggesting that mobile phones apps are a viable tool to access learning and reference materials. The increasing availability of these mobile phones devices and the possibility of accessing the content on the go suggest that there could be a further peak in usage if the internal system in Bern is opened up for mobile phones access. The effect on patient care remains unclear, but usage patterns suggest that electronic access to guidelines is highly appreciated. Mobile phones apps are an emerging tool in health care [24-27], and they are leading to new possibilities, such as applications in patient management, resource distribution, and quality control [28]. Our Web-based system and most of the apps available fall in the category of patient management [28-30]. Some trials investigating the effect of mobile technology have shown that it significantly improves outcomes related to disease management [29].

### Improving Outcome and Cost-Effectiveness

Studies concerned with the implementation of digital solutions have shown variable results with regard to guideline adherence [14,31-33], but the scientific data on this topic are limited. To further develop the CMS to meet the needs of our users, we intend to assess the effect of the implemented systems through frequent administration of questionnaires, and if feasible, an outcome-related study will be considered. Owing to its high usage, ease of use, and low cost, our Web-based repository for health care guidelines and operating procedures could potentially contribute to the digitization of other health care organizations with needs similar to ours [34]. Solutions that can be developed rapidly and implemented easily may be crucial for the survival of organizations in the health care landscape [34].

### Conclusions

We have demonstrated that WordPress is a suitable solution for distributing and managing the internal SOPs and guidelines of 2 tertiary anesthesia departments. Although our study was performed solely in anesthesia departments, implementation in different areas of health care seems feasible. Metadata allow live monitoring and feedback. The systems are cost effective and can be handled from within the department, without depending on third-party support.

## Abbreviations

CMS: content management system
SOP: standard operating procedure
